# Is local alcohol outlet density related to alcohol-related morbidity and mortality in Scottish cities?

**DOI:** 10.1016/j.healthplace.2015.02.014

**Published:** 2015-05

**Authors:** E.A. Richardson, S.E. Hill, R. Mitchell, J. Pearce, N.K. Shortt

**Affiliations:** a Centre for Research on Environment, Society and Health (CRESH), School of GeoSciences, University of Edinburgh, Edinburgh EH8 9XP, UK; b Global Public Health Unit, School of Social and Political Science, University of Edinburgh, EH8 9LD, UK; c Centre for Research on Environment, Society and Health (CRESH), Section of Public Health and Health Policy, Faculty of Medicine, University of Glasgow, Glasgow G12 8RZ, UK

**Keywords:** Alcohol outlets, Drinking, Hospitalization, Mortality, Scotland

## Abstract

Alcohol consumption may be influenced by the local alcohol retailing environment. This study is the first to examine neighbourhood alcohol outlet availability (on- and off-sales outlets) and alcohol-related health outcomes in Scotland. Alcohol-related hospitalisations and deaths were significantly higher in neighbourhoods with higher outlet densities, and off-sales outlets were more important than on-sales outlets. The relationships held for most age groups, including those under the legal minimum drinking age, although were not significant for the youngest legal drinkers (18–25 years). Alcohol-related deaths and hospitalisations were higher in more income-deprived neighbourhoods, and the gradient in deaths (but not hospitalisations) was marginally larger in neighbourhoods with higher off-sales outlet densities. Efforts to reduce alcohol-related harm should consider the potentially important role of the alcohol retail environment.

## Highlights

• Whether alcohol outlet availability influences health in Scotland was unknown
• We assessed relationships with alcohol-related hospitalisations and mortality
• More hospitalisations and deaths occurred in areas with greater outlet availability
• Off-sales outlets were more important for health than on-sales outlets
• Efforts to reduce alcohol harms should consider the alcohol retail environment

## 1 Background

In recent years there has been increasing recognition that place can constrain or enable various health behaviours, including alcohol consumption (Jayne et al., 2010; Pearce et al., 2012). Geographical work has emphasised that a multitude of social, cultural, political and economic factors interact in complex ways to affect alcohol consumption, across spatial scales from the global to the local (Jayne et al., 2008). The importance of geographical context in understanding drinking behaviour is emphasised by the spatial differences in consumption and related health outcomes across Great Britain (Beeston et al., 2014; Leon and McCambridge, 2006). There are various pathways through which place may influence individual behaviours such as alcohol consumption (Bernard et al., 2007; Macintyre and Ellaway, 2000). Various studies attest to the importance of geographical context in drinking behaviours, norms and cultures (Bryden et al., 2013; Holloway et al., 2008; Hughes et al., 2011; Valentine et al., 2008). Alcohol-related health outcomes are also spatially patterned (Emslie and Mitchell, 2009). With alcohol consumption among women and young adolescents increasing in the UK (Meng et al., 2014; Smith and Foxcroft, 2009), and preferred drinking venues changing across the life course (Information Services Division, 2010), the role of place should not be overlooked.

One geographical factor that may influence alcohol consumption is the availability of alcohol retail outlets. Neighbourhood availability of alcohol retailing may influence local consumption patterns and health outcomes through a number of pathways. Greater local availability of alcohol retailers, and increased visibility of their advertising and promotions, can increase the physical availability of alcohol, reduce the prices of alcohol products due to retailer competition, and shape and reinforce local attitudes and norms around drinking behaviours and drunkenness (Livingston et al., 2007; Pasch et al., 2009, 2007). Increased consumption levels may result; for example lower alcohol prices tend to lead to increased consumption (Babor et al., 2003). Indeed, studies indicate that population-wide consumption of alcohol may be higher in neighbourhoods with higher alcohol outlet densities (Ayuka et al., 2014; Bryden et al., 2012). Local alcohol outlet densities have also been linked to acute alcohol-related health problems such as assault and vehicle collision injuries (Gruenewald et al., 2006; LaScala et al., 2001; Livingston, 2008b; Treno et al., 2007). Chronic alcohol-related health problems have received less attention, although these are a more prevalent health consequence of alcohol consumption in people over 35 years of age (Grant et al., 2009). Recent work in the US and Australia indicates that chronic consequences such as cirrhosis and mental disorders are more prevalent in neighbourhoods with higher densities of retail outlets licensed to sell alcohol for consumption off the premises (Pereira et al., 2013; Theall et al., 2009).

Different types of alcohol outlet are likely to encourage distinct types of drinking behaviours and hence may influence health in varying ways. A key distinction is whether the outlet is licensed to sell alcohol for consumption on the premises (‘on-sales’ outlets, such as bars and restaurants) or off the premises (‘off-sales’ outlets, such as convenience stores and supermarkets). While political and media attention has often been concerned with binge drinking in public spaces dominated by on-sales premises, such as city-centre ‘entertainment districts’, less attention has been paid to the less visible drinking conducted at home, with alcohol purchased from off-sales premises (Holloway et al., 2008). Indeed, Forsyth and Davidson (2010) argue that off-sales outlets have greater potential for alcohol-related harm than on-sales premises. In an Australian study, Livingston (2011) compared the relationship of acute and chronic alcohol-related health outcomes with local densities of off-sales and on-sales outlets and found that chronic health outcomes (mental and behavioural) were strongly related to off-sales outlet densities. This finding might be because ‘problematic’ drinkers seek to acquire alcohol from the cheapest available sources (i.e., for off-premise consumption). Moreover, off-sales outlets tend to be disproportionately concentrated in areas of socioeconomic disadvantage (Ellaway et al., 2010; Hay et al., 2009; Livingston, 2012; Romley et al., 2007). In contrast, on-sales outlets are not so clearly patterned, and in some cases are more concentrated in more affluent areas (Ellaway et al., 2010; Hay et al., 2009; Livingston, 2012).

Scotland has one of the highest levels of alcohol-related harm in Western Europe (Beeston et al., 2013). In the 1990s cirrhosis mortality declined in many European countries but increased steeply in Scotland, leading to calls for action to halt the alarming trend (Leon and McCambridge, 2006). Scotland has the highest rate of alcohol-related mortality in the UK (Breakwell et al., 2007); male alcohol-related mortality rates in Scotland were more than double those in England and Wales for most years in the last two decades (Beeston et al., 2013). In Scotland one in every 20 deaths and one in every 20 hospital episodes is attributable to alcohol (Grant et al., 2009). The negative consequences of alcohol use were conservatively estimated to cost Scottish society £3.6 billion in 2007, of which £268 million were incurred by the National Health Service (NHS)(Scottish Government, 2010).

Scotland also has a marked social gradient in both hospitalisations and deaths due to alcohol (Beeston et al., 2013), contributing to widening socioeconomic health inequalities (Audit Scotland, 2012). While alcohol consumption is high across all deciles of deprivation, Scots living in the most deprived neighbourhoods have slightly higher levels of harmful consumption but almost 10 times the rate of both hospitalisation and death due to alcohol, compared with those living in the least deprived areas (Beeston et al., 2013). Of all health inequalities monitored by the Scottish Government the highest are seen for alcohol-related deaths in 45–74 year olds (Scottish Government, 2013).

Given the magnitude of the health, social and economic costs of alcohol-related harm in Scotland, and that the health burden is falling disproportionately upon the most socially disadvantaged communities, there is an urgent need for a better understanding of potential policy levers for reducing high levels of alcohol consumption (Scottish Government, 2009). Scotland׳s current alcohol strategy relies heavily on education and early intervention, but also recognises the need to address alcohol availability – particularly via off-sales, which are seen as playing a growing role in unhealthy levels of consumption (Scottish Government, 2009). In 2012 the Scottish Parliament made a significant step in this regard, passing legislation introducing a minimum unit price (MUP) for alcohol sales (Forsyth et al., 2014). This initiative met with strong opposition and a legal challenge from the alcohol industry (led by the Scotch Whisky Association), and plans for implementation of MUP currently remain on hold (Forsyth et al., 2014).

The current Scottish study aimed to address the absence of neighbourhood-level work on alcohol outlet density and chronic health outcomes from countries other than the US or Australia. To date, little is known about the role of the alcohol retail environment in alcohol consumption and health in Scotland. In Australia and the US different urbanisation patterns have tended to result in lower population densities and higher levels of social and ethnic segregation. It might be anticipated that in these national contexts this may result in a lower density of outlets but with a greater social discrepancy due to the concentration of targeted consumers and alternative land use planning strategies. The only Scottish research in this area is limited to research on adolescents in Glasgow (Young et al., 2013). Our study extends the research by considering a larger adult population living in the main urban areas across the country.

The aims of this study were to: (a) examine the association between neighbourhood densities of on-sales and off-sales outlets and alcohol-related morbidity and mortality in Scottish cities; (b) determine whether the relationship differed by age group and sex; and (c) assess whether socioeconomic gradients in alcohol-related health outcomes differed by level of outlet density. In addition to investigating relationships with all alcohol-related health outcomes combined, we also focused on cirrhosis cases specifically as an indicator of chronic alcohol-related harm. We hypothesised that cirrhosis would be more strongly related to off-sales than on-sales outlet densities, because prolonged excessive drinking is likely to be facilitated by cheaper alcohol, such as that sold for off-premise consumption (Livingston, 2011). We expected different relationships by age group and sex based on well-documented differences in the drinking venues and patterns of these groups (Information Services Division, 2010; Valentine et al., 2010; Young et al., 2013) and different disease latency. Alcohol-related health outcomes in young legal drinkers were expected to be less related to their local alcohol environment than those in older drinkers, because younger people׳s harmful drinking often occurs in central entertainment districts rather than their local neighbourhood (Hayward and Hobbs, 2007). Alcohol-related health outcomes in drinkers under the UK׳s legal minimum drinking age (18 years) were expected to be more strongly related to off-sales than on-sales outlet densities, due to the majority of their alcohol purchases being made from off-sales outlets (Information Services Division, 2009). Differences in these relationships between males and females were expected to be larger in older age groups, based on evidence that young women׳s drinking habits have become increasingly similar to young men׳s (Valentine et al., 2010).

## 2 Data and methods

### 2.1 Design

Alcohol outlet density measures were calculated for the Scottish ‘datazone’ administrative geography. Datazones are the Scottish Government׳s smallest geographical unit for the dissemination of administrative data, and were designed to contain populations between 500 and 1000. We then assessed the relationship of outlet density with alcohol-related hospitalisation and mortality, after accounting for relevant covariates.

### 2.2 Alcohol outlet availability

Postcode-referenced records of licensed premises (correct as of 2008) were obtained from the Liquor Licensing Boards of the four largest cities in Scotland: Glasgow, Edinburgh, Aberdeen and Dundee. Resource constraints precluded collecting outlet data for the whole of Scotland (over 30 licensing boards, with no central register), hence we selected the largest concentrations of outlets and population. The records distinguished premises licensed to sell alcohol for consumption either off or on the premises (off-sales and on-sales premises, respectively). A separate on and off-sales licence was also granted in some cases, but we were advised that sales from these premises would be largely on-premise (Edinburgh licensing board, pers. comm.), hence they were treated as on-sales outlets.

Outlet density measures are often calculated for administrative geographies such as zip codes, census tracts or postcodes (Freisthler et al., 2008; Livingston, 2011; Theall et al., 2009; Treno et al., 2007). Implicit in such measures are the assumptions that (a) the population is evenly distributed across each geographical unit and (b) residents are unaffected by outlets outside of these artificially-imposed boundaries. We also utilised an administrative geography – datazones – but (a) calculated our density measure for the population centre of each unit, in order to capture the alcohol environment that the majority of the population were exposed to, and (b) quantified outlet density within a radius around this point, ensuring that the measure was not constrained to datazone boundaries. We used the technique of kernel density estimation (‘KDE’, see Carlos et al., 2010) to generate our density measure because it uses distance-decay weighting (outlets closer to the datazone׳s population centre were weighted higher than those further away), and we conceptualised that outlets closer to the population may have greater influence on consumption and health. KDE measures of alcohol outlet density have been used in studies of the distribution of alcohol outlets (Berke et al., 2010) and of relationships with health outcomes (Major et al., 2014; Pearson et al., 2014).

We created a measure of alcohol outlet density per unit area rather than population, on the basis that an individual׳s alcohol consumption is more likely to be influenced by the absolute physical availability of an outlet rather than how many people the outlet is shared between. An alcohol outlet density surface was then produced for each city using KDE. First, the cities were divided into 50×50 m^2^ ‘kernels’. Second, for each kernel the number and proximity of outlets within a 1 km search radius were used to calculate a density measure, with outlets nearer the kernel being given greater weight. Density surfaces were produced for on-sales and off-sales premises, providing a proximity-weighted estimate of the density of each outlet type per km^2^ (termed “proximity-weighted outlets per km^2^” hereafter, as the weighting process means that the values differ from standard density measures). The outlet data were analysed using ArcMap 9.3 Geographic Information System (GIS) software (ESRI, Redlands, CA).

We extracted outlet density values at the population-weighted centroid of each datazone in the four licensing board areas. Datazones (mean 2001 population 821, range 499–2692) were the smallest spatial units for which sufficiently large health outcome counts could be obtained, to enable reliable statistical modelling. The four areas were not adjacent and we did not have outlet data for surrounding licensing board areas, hence potential density underestimation was avoided by restricting our sample to the 1360 datazones with population-weighted centroids at least 1 km from the landward edge of each licensing board area.

### 2.3 Health outcome data

Anonymised individual occurrences of alcohol-related hospitalisation and death between 2000 and 2009 were obtained from NHS Scotland׳s Information Services Division (ISD). Each record included age group (0–17, 18–24, 25–29, 30–49, 50–59, 60–74 and 75 plus) and sex. The ISD definition of alcohol-related health outcomes (Information Services Division, 2009) captures conditions that are wholly attributable to alcohol (ICD10 codes E24.4, E51.2, F10, G31.2, G62.1, G72.1, I42.6, K29.2, K70, K86.0, O35.4, P04.3, Q86.0, R78.0, T51.0, T51.1, T51.9, X45, X65, Y15, Y57.3, Y90, Y91, Z50.2, Z71.4, Z72.1), and combines acute and chronic consequences. In 2007/08, mental and behavioural diagnoses were the most common alcohol-related cause of hospitalisation (71%), followed by cirrhosis (16%) and alcohol poisoning (11%) (Information Services Division, 2009). We also extracted hospitalisations and deaths due to cirrhosis (ICD10 K70) alone, to capture a wholly chronic effect of alcohol use. As our interest was in population response to outlet densities rather than a cumulative measure of impact on individuals we included only the first hospitalisation for each health outcome for each individual. Hospitalisations and deaths with either a main or supplementary diagnosis of interest were included.

### 2.4 Area-level covariates

For each datazone we extracted population counts (2001) by age group and sex, and a measure of area-level deprivation. The Scottish Index of Multiple Deprivation (SIMD) quantifies multiple deprivation on seven domains – employment, income, crime, housing, health, education and access – at the datazone level. The overall score includes an aggregate measure of alcohol-related hospitalisations, hence we selected the SIMD׳s Income Deprivation domain from 2006. This domain quantifies the percentage of the population in receipt of income support benefits.

### 2.5 Data linkage

Alcohol outlet densities and the covariates were appended to each record by ISD, based on the individual׳s datazone of residence at the time. ISD then removed the datazone identifiers before providing the linked dataset. The release of individual health data in this form was given ethical approval by the NHS Privacy Advisory Committee.

### 2.6 Temporal coverage

The health data covered 2000–2009, to ensure sufficient counts, although the alcohol outlet data were collected in 2008. We assessed whether 2008 data would adequately capture the alcohol environment over a wider period by comparing with outlet data collected for the same cities in 2012 (Shortt et al., In preparation). We found minimal change over the four years: 88% of the datazones had identical numbers of on-sales premises in the two datasets (89% for off-sales licences).

### 2.7 Analyses

The relationships between alcohol outlet density and health outcomes (counts of hospitalisations or deaths per datazone) were modelled using Poisson regression in Stata/IC 11.1 (StataCorp, College Station, TX). We modelled counts rather than rates because the datazones had small populations, which meant that rates would have been highly sensitive to small differences in the numerator. Baseline models were adjusted for age group, sex, datazone-level income deprivation rate, and city. City was included in the models to check whether the relationships varied between the cities. Age- and sex-specific population counts (from 2001 census) were used as the denominator. Subsequent models were stratified by sex and age group and by outlet density quartiles. Incidence rate ratios were calculated to represent the relative increase in the incidence rate of the health outcome associated with an interquartile range (IQR) increase in alcohol outlet density. The IQRs – the difference between the 25th and 75th percentiles – were 11.9 proximity-weighted outlets per km^2^ for on-sales and 10.6 for off-sales. On-sales and off-sales outlet density measures were highly correlated (*r*>0.95, *p*<0.001), hence could not be included in the same models because of multicollinearity issues. Separate models were run for on- and off-sales densities, and Akaike׳s Information Criterion (AIC) values were compared to assess which of the models produced the best fit to the data.

Such analyses may be biased due to non-independence of the geographical units, or ‘spatial autocorrelation’, hence it is typical to run spatial error models to adjust for this. However, due to the sensitive nature of these small-area data the geographic identifiers had been removed by the data provider, precluding any assessment of spatial autocorrelation. We note, however, that even where alcohol outlet and harm data have been found to be significantly autocorrelated the corrected results are often not substantively different to those without a spatial error term (Livingston, 2008a, 2010; Tatlow et al., 2000).

## 3 Results

### 3.1 Descriptive statistics

Descriptive statistics for the 1360 datazones included in the study are given in Table 1. For both alcohol outlet types, densities were highest on average in Edinburgh, and lowest in Dundee and Aberdeen.

The included datazones had a combined population of 1.1 million in 2001, or 22% of the Scottish population. From this population a total of 45,444 individuals were hospitalised for alcohol-related conditions between 2000 and 2009, of whom 3970 (9%) were hospitalised for cirrhosis. Alcohol-related deaths numbered 7064, of which 3952 (56%) were attributable to cirrhosis. Nil or low counts restricted the age groups that could be included in the models for each health outcome: all ages for alcohol-related hospitalisations, 18+ for cirrhosis hospitalisations, and 25+ for all alcohol-related or cirrhosis deaths.

### 3.2 Are alcohol-related health outcomes related to outlet densities?

Higher densities of on- and off-sales outlets were related to significantly higher incidence of all alcohol-related health outcomes (Tables 2 and 3). Effect sizes were larger for off-sales than on-sales density, and AIC values indicated that the off-sales density models produced the best fit to the data. Effect sizes were also larger for mortality than for hospitalisation. An IQR increase in off-sales outlet density was associated with higher incidence of all alcohol-related conditions in general (8% higher hospitalisation, 19% higher mortality), and cirrhosis (11% higher hospitalisation, 15% higher mortality). Incidence of all outcomes was significantly lower for females than males, and at younger ages. Each percentage-point increase in income deprivation was associated with an average 4% increase in hospitalisation or mortality.

### 3.3 Do relationships between outlet densities and health vary by age and sex?

Stratified models revealed clear variation in the relationships between outlet densities and health outcomes by age and sex (Table 4). On- and off-sales densities produced similarly-patterned associations, although effect sizes were higher for off-sales outlets and AIC values again indicated a better fit to the data for these models.

Underage (<18 years) hospitalisations for all alcohol-related outcomes were significantly related to outlet densities, and the associations were strongest for females. An IQR increase in off-sales outlet density was associated with a 19% increase in alcohol-related hospitalisations for females (8% for males). For females the relationship with alcohol-related hospitalisation was strongest in this age group.

All alcohol-related hospitalisations among 18–24 year olds were lower in areas with higher outlet densities. The relationship was significant for males (*p*<0.001) such that an IQR increase in densities of off-sales outlets was associated with 7% *fewer* male alcohol-related hospitalisations. No significant associations with hospitalisation were found for 25–29 year old males or females, but rates of all alcohol-related mortality for 25–29 year old males were significantly higher in neighbourhoods with higher outlet densities.

Cirrhosis outcomes, particularly deaths, were most strongly related to outlet densities for the older age groups. Cirrhosis hospitalisation and death in under 30 year olds was rare – 70 and 31 cases respectively – hence the absence of a relationship with area-level outlet density is not surprising.

Clear patterning by sex was only observed for all alcohol-related hospitalisations; in groups 30 years old and over, hospitalisation rates were more strongly related to local outlet densities for males than for females.

### 3.4 Is outlet density related to socioeconomic gradients in alcohol-related health?

We used interaction models to investigate whether the socioeconomic gradients in alcohol-related health outcomes were affected by outlet densities. Continuous-by-continuous interaction models showed small but significant positive interactions for off-sales outlet densities in the relationship between income deprivation and mortality from all alcohol-related conditions combined (*p*=0.024) or cirrhosis (*p*=0.048), but neither for hospitalisations, nor for on-sales densities. Hence the socioeconomic gradients in alcohol-related and cirrhosis deaths were slightly steeper in datazones with higher densities of off-sales premises. In datazones with below-average off-sales outlet densities, for example, a 1% increase in income deprivation rate was associated with a 4.2% increase in all alcohol-related deaths, whereas the equivalent figure was 4.6% for datazones with higher than average densities.

## 4 Discussion

There is growing recognition amongst researchers and policymakers that geographical context is important for understanding health behaviours, including alcohol consumption. Understanding these processes is not only an academic concern but also offers options to policymakers tasked with reducing the societal burden of alcohol-related harm and related health inequalities. A multitude of place-based factors have been implicated in understanding patterns of alcohol consumption including the local availability of alcohol retailing. This study is the first in Scotland to examine the relationship between alcohol retailing and alcohol-related health outcomes amongst adults. We distinguished between off-sales and on-sales premises, responding to concerns that previous work has been biased towards public drinking spaces, while off-sales outlets have greater potential for producing harmful drinking behaviours, particularly in underage groups (Forsyth and Davidson, 2010; Holloway et al., 2008).

We found that alcohol outlet densities were associated with alcohol-related health outcomes in Scottish cities; rates of hospitalisation and mortality from all alcohol-related outcomes in general, and cirrhosis in particular, were significantly higher in populations of neighbourhoods with higher alcohol outlet densities. Strong relationships were also found for underage drinkers, particularly females. Income-related gradients in alcohol-related mortality were marginally larger in neighbourhoods with higher off-sales outlet densities.

Our indicator of chronic alcohol-related harm – cirrhosis – was more strongly related to off-sales than on-sales densities, as predicted, but this was also the case for all alcohol-related health outcomes (combining chronic and acute harms). The relative importance of on- and off-sales densities to the relationship could not be assessed, although model diagnostics suggested that on-sales densities were acting as a proxy for off-sales densities. This supports the claim of Forsyth and Davidson (2010) that off-sales outlets have the greatest potential for alcohol-related harm, due to their cheaper product, accessibility for under-age drinkers, large volumes obtainable, and absence of control over the final recipient. Other evidence shows the growing importance of off-premise consumption of alcohol. While on-sales premises dominated the alcohol retail environment numerically in our study (73% of total outlets), off-sales outlets account for a more than 60% and rising share of alcohol sales in Scotland by volume (Information Services Division, 2010). Indeed, drinking at home has become more affordable and more socially accessible in recent years (Foster et al., 2010; Holloway et al., 2008). In addition, we suggest that local availability of off-sales outlets may have a stronger link to neighbourhood-level alcohol consumption and harms than on-sales outlets because of the convenience constraints of carrying the alcohol purchased to another location, often the home, for later consumption. Our findings, coupled with increased alcohol sales from off-sales outlets, give weight to claims of a misplaced policy focus on the night-time economy (Holloway et al., 2008), and challenge us to reconsider the spatial framing of drinking and related harms from public to private space.

Contrasting our findings for deaths and hospitalisations may shed light on the relative importance of the alcohol environment for chronic versus acute alcohol-related harms. Outlet densities were more strongly related to alcohol-related deaths than hospitalisations. Most alcohol-related deaths were due to chronic causes (56% were cirrhosis, and an unknown additional proportion were due to alcoholic gastritis, alcoholic cardiomyopathy, and other chronic disease) hence the finding suggests that the local alcohol environment – and most likely off-sales rather than on-sales outlets – is particularly important for the long-term excessive drinking that initiates the development of chronic alcohol-related illness. High densities of alcohol outlets may simultaneously increase the availability of alcohol and normalise alcohol consumption behaviours for local communities.

There is growing concern about increasing levels of alcohol consumption by young people which have led to increases in chronic alcohol-related illness at younger ages (Chief Medical Officer, 2001). Outlets selling alcohol for consumption off the premises are likely to be important sources of alcohol for underage drinkers (Forsyth and Davidson, 2010). Our finding that alcohol-related hospitalisation of underage drinkers was related to off-sales densities supports this claim. Alcohol outlet densities have been linked with under 18 year olds׳ drinking (Chen et al., 2010, 2009; Young et al., 2013) and health outcomes inflicted on children by an adult (assault or other maltreatment) (Alaniz et al., 1998; Freisthler et al., 2008, 2004), but we are not aware of other work that has found links with health consequences of under 18 year olds׳ own drinking. Although underage drinkers more frequently source alcohol from social contacts rather than directly through retail outlets (Hearst et al., 2007; Information Services Division, 2010), both routes may be influenced by the physical availability of alcohol outlets. Moreover, alcohol purchases by underage customers are more successful if there are similar retail outlets nearby (Freisthler et al., 2003).

We expected the local alcohol environment to have least importance for alcohol-related health consequences of young legal drinkers because the riskiest drinking behaviours among this group may occur in out-of-neighbourhood entertainment districts (clusters of pubs and clubs) (Hayward and Hobbs, 2007). Accordingly, we found no relationship between outlet densities and alcohol-related hospitalisations for 25–29 year old males and females and 18–24 year old females, but found a surprising negative relationship for the youngest legal male drinkers (18–24). A possible explanation is that in residential neighbourhoods with higher densities of outlets, perhaps representing a sufficient choice of alcohol sources, young people may be less inclined to visit out-of-neighbourhood outlet aggregations, and may be less likely to engage in the risky drinking practices that such concentrations of young drinkers and alcohol sources can encourage. That alcohol-related deaths in 25–29 year old males were positively related to outlet densities complicates this story, but may be attributable to the heaviest drinkers being able to source large quantities of cheap alcohol most easily from local outlets (particularly off-sales outlets).

We also investigated whether the associations between neighbourhood outlet densities and the health outcomes varied by social group and hence might be a potential factor in explaining health inequalities. An Australian study attributed widening socioeconomic inequalities in cirrhosis mortality to increasing availability of alcohol over time (Najman et al., 2007). In our study, income-related gradients in deaths (but not hospitalisations) from cirrhosis and all alcohol-related outcomes in general were slightly wider in neighbourhoods with higher densities of off-sales outlets (but not on-sales). A possible mechanism is that consumption of alcohol at harmful levels is most prevalent among low income men and women (Information Services Division, 2010), and drinking among the heaviest drinkers is most sensitive to increases in alcohol availability (Makela, 2002). Our findings suggest that increased densities of off-sales alcohol outlets may result in wider socioeconomic inequalities in alcohol consumption and its health consequences.

Our study had limitations. First, whilst our outlet density measures indicate the neighbourhood availability of alcohol retailing, we were not able to include other attributes of retailing that may influence alcohol consumption. Future work could usefully include information on prices, trading hours, venue type and on-sales capacity. Second, our kernel density measures were necessarily based on ‘as-the-crow-flies’ radii, which could not account for barriers to movement. While this meant that not all outlets captured by the measure could be accessed on a 1 km journey on roadways or paths the proximity-weighting applied meant that the measure reflected the density of closer outlets more than those further away. Third, to ensure accurate representation of outlet density we excluded areas on the periphery of each licensing board area – largely suburban areas with low outlet densities – which will have introduced some bias towards inner city locations. Fourth, exposure misclassification, a common issue for ecological studies, was inevitable because we considered only the outlet densities within the neighbourhood of residence at the time of hospitalisation or death. In reality individuals move between areas with different alcohol outlet densities (over daily and longer time periods). Future work could usefully incorporate a variety of geographical settings to which individuals are exposed over the course of their daily lives (e.g. residential neighbourhood, workplace and recreational settings). Work in the field of physical activity that tracks individual ‘activity spaces’, often using GPS technology, offers significant potential to research on alcohol environments. Longer term – and often longer distance – residential movements could also be studied using longitudinal datasets. Nonetheless, the fact that outlet densities in the place of residence at the time of hospitalisation or death were positively related to cirrhosis rates is noteworthy, given the long lag period of the disease. The alcohol retail environment in the individual׳s place of residence at the time of their hospitalisation or death might therefore be indicative of the alcohol environment they had experienced during the development of the disease. Fifth, causal inference is not possible because of the cross-sectional study design. Longitudinal analysis is required to ascertain the mechanisms underlying the relationships we found, recognising that supply and demand dynamics interact to shape alcohol outlet availability and alcohol consumption patterns (Gruenewald, 2008).

Our findings have direct implications for alcohol policy in Scotland. In particular, the association between off-sales alcohol outlet density and alcohol-related hospitalisations and deaths highlights the potential for MUP (which primarily affects off-sales) to reduce alcohol-related harm at a population level. This potential is particularly relevant for efforts to reduce harmful alcohol consumption in more deprived neighbourhoods, where the impact of MUP on off-sales is likely to be most pronounced (Forsyth et al., 2014). The strength of the relationship between outlet density and alcohol-related hospitalisations of underage drinkers also highlights the need for greater efforts to reduce alcohol availability to this group.

Reducing alcohol outlet densities – particularly of off-sales outlets – could potentially help to reduce population-wide alcohol-related harm. Alcohol licensing regulations offer one possible mechanism for reducing outlet density, as recognised in WHO Europe׳s action plan on alcohol (World Health Organization, 2012). Scotland׳s 2005 Licensing Act includes provision for Licensing Boards to take account of “Protecting and improving public health” in making licensing decisions (Scottish Parliament, 2007, p9), although there is little evidence that this provision is often utilised.

## 5 Conclusions

Our study helps to further understanding of how social and spatial factors influence alcohol issues. Health problems attributable to alcohol were more prevalent in neighbourhoods with more alcohol outlets, which may indicate that outlet availability influences alcohol consumption. Availability of off-sales outlets appeared to drive these relationships, in line with the increasing dominance of off-premise drinking in UK. Alcohol-related hospitalisations of those under the legal minimum drinking age were also related to outlet densities, suggesting a need for greater enforcement of alcohol retail legislation. However, among the youngest legal drinkers alcohol-related health outcomes were somewhat decoupled from the residential alcohol environment. Tackling alcohol-related problems in this subgroup of the population is likely to require a wider focus, as alcohol outlets outside the local neighbourhood may be more important. Efforts to reduce alcohol-related harm should consider the role of the alcohol retail environment.

## Figures and Tables

**Table 1:** Descriptive statistics for the 1360 included datazones: combined and by city.

	All	Aberdeen	Dundee	Edinburgh	Glasgow
N included datazones	1360	235	136	527	462
Total included population (2001)	1,112,956	182,557	112,827	429,586	387,986
Datazone characteristics (mean(SD))					
Population (2001)	821 (166)	792 (155)	830 (141)	816 (180)	840 (158)
% income deprived (2006)	16.9 (13.6)	11.7 (9.9)	19.9 (11.4)	11.4 (11.0)	24.9 (14.2)
Proximity-weighted alcohol outlet density per km^2^ at PWC:					
on-sales outlets mean (SD)	13.4 (23.0)	9.6 (21.6)	9.7 (12.9)	16.5 (27.4)	13.0 (20.0)
*range*	(*0–187*)	(*0–125*)	(*0–78*)	(*0–187*)	(*0–151*)
off-sales outlets mean (SD)	11.9 (15.0)	7.9 (10.6)	8.8 (8.7)	15.3 (19.5)	10.9 (11.0)
*range*	(*0–127*)	(*0–59*)	(*1–47*)	(*0–127*)	(*0–83*)
Health outcome counts per datazone (mean (SD)) (2000–2009 aggregated)					
Hospitalisation					
Cirrhosis	2.9 (2.9)	1.9 (1.9)	3.0 (2.2)	2.0 (2.2)	4.4 (3.5)
Other alcohol-related conditions	30.5 (23.1)	30.6 (20.6)	25.1 (14.0)	22.5 (16.9)	41.2 (27.8)
Death					
Cirrhosis	2.9 (3.2)	1.5 (1.7)	2.5 (2.2)	2.2 (2.3)	4.6 (4.0)
Other alcohol-related conditions	2.3 (2.7)	1.4 (1.7)	3.0 (2.6)	2.0 (2.4)	2.8 (3.2)

PWC=population-weighted centroid; SD=standard deviation.

**Table 2:** The relationship between on-sales alcohol outlet density and incidence of alcohol-related hospitalisation or death^a^.

	All alcohol-related health outcomes		Cirrhosis	
	Hospitalisation	Death	Hospitalisation	Death
On-sales outlet density (IRR per IQR increase)	1.05 (1.04–1.06)⁎⁎⁎	1.12 (1.10–1.14)⁎⁎⁎	1.06 (1.04–1.09)⁎⁎⁎	1.09 (1.07–1.11)⁎⁎⁎
Sex				
Male	1.00	1.00	1.00	1.00
Female	0.40 (0.39–0.41)⁎⁎⁎	0.32 (0.31–0.34)⁎⁎⁎	0.41 (0.38–0.44)⁎⁎⁎	0.39 (0.36–0.41)⁎⁎⁎
Age group				
0–17	0.18 (0.17–0.19)⁎⁎⁎	-	-	-
18–24	0.81 (0.78–0.84)⁎⁎⁎	0.02 (0.01–0.03)⁎⁎⁎	0.03 (0.02–0.05)⁎⁎⁎	-
25–29	0.68 (0.65–0.71)⁎⁎⁎	0.11 (0.08–0.14)⁎⁎⁎	0.13 (0.10–0.18)⁎⁎⁎	0.09 (0.06–0.13)⁎⁎⁎
30–49	1.00	1.00	1.00	1.00
50–59	1.38 (1.33–1.42)⁎⁎⁎	2.91 (2.72–3.12)⁎⁎⁎	2.40 (2.22–2.60)⁎⁎⁎	2.87 (2.63–3.12)⁎⁎⁎
60–74	1.25 (1.21–1.28)⁎⁎⁎	3.05 (2.85–3.26)⁎⁎⁎	1.90 (1.75–2.06)⁎⁎⁎	2.80 (2.57–3.04)⁎⁎⁎
75 plus	0.86 (0.82–0.90)⁎⁎⁎	1.78 (1.62–1.96)⁎⁎⁎	0.79 (0.69–0.92)⁎⁎	1.60 (1.42–1.81)⁎⁎⁎
Datazone deprivation				
% income deprived	1.05 (1.04–1.05)⁎⁎⁎	1.04 (1.04–1.05)⁎⁎⁎	1.04 (1.04–1.04)⁎⁎⁎	1.04 (1.04–1.05)⁎⁎⁎
City				
Glasgow	1.00	1.00	1.00	1.00
Aberdeen	1.40 (1.32–1.49)⁎⁎⁎	0.82 (0.73–0.93)⁎⁎	0.81 (0.72–0.92)⁎⁎	0.64 (0.55–0.73)⁎⁎⁎
Dundee	0.84 (0.78–0.90)⁎⁎⁎	0.98 (0.87–1.11)	0.83 (0.73–0.94)⁎⁎	0.67 (0.58–0.77)⁎⁎⁎
Edinburgh	1.01 (0.96–1.06)	1.02 (0.93–1.12)	0.79 (0.72–0.87)⁎⁎⁎	0.83 (0.75–0.92)⁎⁎⁎

IRR, incidence rate ratio; IQR, interquartile range (11.9 proximity-weighted on-sales outlets per km^2^).

⁎⁎ 0.001<*p*≤0.01.

⁎⁎⁎ *p*<0.001.

a Models adjusted for age group, sex, datazone level income deprivation, and city.

**Table 3:** The relationship between off-sales alcohol outlet density and incidence of alcohol-related hospitalisation or death^a^.

	All alcohol-related health outcomes		Cirrhosis	
	Hospitalisation	Death	Hospitalisation	Death
Off-sales outlet density (IRR per IQR increase)	1.08 (1.07–1.10)⁎⁎⁎	1.19 (1.16–1.22)⁎⁎⁎	1.11 (1.08–1.14)⁎⁎⁎	1.15 (1.11–1.18)⁎⁎⁎
Sex				
Male	1.00	1.00	1.00	1.00
Female	0.40 (0.39–0.41)⁎⁎⁎	0.32 (0.31–0.34)⁎⁎⁎	0.41 (0.38–0.44)⁎⁎⁎	0.39 (0.36–0.41)⁎⁎⁎
Age group				
0–17	0.18 (0.17–0.19)⁎⁎⁎	-	-	-
18–24	0.81 (0.78–0.84)⁎⁎⁎	-	0.03 (0.02–0.05)⁎⁎⁎	-
25–29	0.68 (0.65–0.71)⁎⁎⁎	0.11 (0.08–0.14)⁎⁎⁎	0.13 (0.10–0.17)⁎⁎⁎	0.09 (0.06–0.13)⁎⁎⁎
30–49	1.00	1.00	1.00	1.00
50–59	1.38 (1.34–1.42)⁎⁎⁎	2.93 (2.73–3.14)⁎⁎⁎	2.41 (2.22–2.61)⁎⁎⁎	2.88 (2.64–3.14)⁎⁎⁎
60–74	1.25 (1.21–1.29)⁎⁎⁎	3.06 (2.87–3.28)⁎⁎⁎	1.91 (1.76–2.07)⁎⁎⁎	2.80 (2.58–3.05)⁎⁎⁎
75 plus	0.86 (0.82–0.90)⁎⁎⁎	1.79 (1.62–1.97)⁎⁎⁎	0.80 (0.69–0.92)⁎⁎	1.61 (1.42–1.82)⁎⁎⁎
Datazone deprivation				
% income deprived	1.04 (1.04–1.05)⁎⁎⁎	1.04 (1.04–1.05)⁎⁎⁎	1.04 (1.04–1.04)⁎⁎⁎	1.04 (1.04–1.04)⁎⁎⁎
City				
Glasgow	1.00	1.00	1.00	1.00
Aberdeen	1.41 (1.33–1.50)⁎⁎⁎	0.83 (0.74–0.93)⁎⁎	0.82 (0.73–0.93)⁎⁎	0.64 (0.56–0.74)⁎⁎⁎
Dundee	0.84 (0.78–0.90)⁎⁎⁎	0.98 (0.87–1.10)	0.83 (0.73–0.94)⁎⁎	0.66 (0.58–0.77)⁎⁎⁎
Edinburgh	0.98 (0.94–1.04)	0.97 (0.89–1.06)	0.77 (0.70–0.84)⁎⁎⁎	0.79 (0.72–0.88)⁎⁎⁎

IRR, incidence rate ratio; IQR, interquartile range (10.6 proximity-weighted off-sales outlets per km^2^).

⁎⁎ 0.001<*p*≤0.01.

⁎⁎⁎ *p*<0.001.

a Models adjusted for age group, sex, datazone level income deprivation, and city.

**Table 4:** The increase in the health outcome rate associated with an interquartile range^a^ increase in on-sales and off-sales outlet densities, stratified by sex and age group^b^. Incidence rate ratios and 95% confidence intervals are given.

Alcohol-related condition		Age group	On-sales outlet density		Off-sales outlet density	
			Male	Female	Male	Female
All	Hospitalisation	0–17	1.05 (1.01–1.11)⁎	1.13 (1.09–1.18)⁎⁎⁎	1.08 (1.01–1.15)⁎	1.19 (1.12–1.26)⁎⁎⁎
		18–24	0.95 (0.93–0.97)⁎⁎⁎	0.98 (0.96–1.01)	0.93 (0.90–0.96)⁎⁎⁎	0.97 (0.94–1.01)
		25–29	0.99 (0.96–1.01)	1.00 (0.97–1.03)	0.97 (0.94–1.01)	1.00 (0.95–1.04)
		30–49	1.09 (1.07–1.11)⁎⁎⁎	1.07 (1.05–1.09)⁎⁎⁎	1.14 (1.11–1.16)⁎⁎⁎	1.10 (1.07–1.13)⁎⁎⁎
		50–59	1.13 (1.11–1.16)⁎⁎⁎	1.10 (1.07–1.13)⁎⁎⁎	1.22 (1.18–1.25)⁎⁎⁎	1.15 (1.11–1.19)⁎⁎⁎
		60–74	1.12 (1.10–1.14)⁎⁎⁎	1.06 (1.03–1.09)⁎⁎⁎	1.19 (1.17–1.22)⁎⁎⁎	1.10 (1.06–1.14)⁎⁎⁎
		75 plus	1.08 (1.06–1.11)⁎⁎⁎	1.03 (0.98–1.08)	1.14 (1.09–1.18)⁎⁎⁎	1.05 (0.98–1.12)
	Death	25–29	1.16 (1.05–1.29)⁎⁎	0.68 (0.39–1.19)	1.22 (1.04–1.42)⁎	0.65 (0.36–1.19)
		30–49	1.10 (1.07–1.14)⁎⁎⁎	1.13 (1.08–1.18)⁎⁎⁎	1.16 (1.11–1.21)⁎⁎⁎	1.21 (1.14–1.28)⁎⁎⁎
		50–59	1.16 (1.13–1.20)⁎⁎⁎	1.12 (1.07–1.18)⁎⁎⁎	1.27 (1.22–1.32)⁎⁎⁎	1.19 (1.12–1.27)⁎⁎⁎
		60–74	1.15 (1.12–1.18)⁎⁎⁎	1.05 (0.99–1.11)	1.24 (1.19–1.29)⁎⁎⁎	1.08 (1.00–1.16)⁎
		75 plus	1.06 (1.01–1.12)⁎	1.09 (1.01–1.18)⁎	1.11 (1.03–1.19)⁎⁎	1.13 (1.02–1.26)⁎
Cirrhosis	Hospitalisation	18–24	0.74 (0.37–0.47)	0.99 (0.70–1.40)	0.72 (0.32–1.62)	0.96 (0.56–1.63)
		25–29	1.08 (0.94–1.24)	0.46 (0.19–1.14)	1.11 (0.91–1.36)	0.39 (0.15–1.01)
		30–49	1.05 (1.01–1.09)⁎	1.11 (1.06–1.16)⁎⁎⁎	1.09 (1.03–1.16)⁎⁎	1.16 (1.08–1.24)⁎⁎⁎
		50–59	1.10 (1.06–1.14)⁎⁎⁎	1.09 (1.03–1.15)⁎⁎	1.18 (1.12–1.24)⁎⁎⁎	1.13 (1.05–1.22)⁎⁎
		60–74	1.05 (1.01–1.09)⁎	1.08 (1.02–1.14)⁎	1.09 (1.03–1.16)⁎⁎	1.13 (1.05–1.23)⁎⁎
		75 plus	1.02 (0.93–1.13)	1.08 (0.96–1.22)	1.03 (0.89–1.19)	1.19 (1.02–1.39)⁎
	Death	25–29	1.01 (0.79–1.28)	0.53 (0.22–1.27)	0.92 (0.61–1.39)	0.46 (0.18–1.20)
		30–49	1.07 (1.03–1.11)⁎⁎	1.13 (1.07–1.19)⁎⁎⁎	1.11 (1.04–1.18)⁎⁎	1.21 (1.13–1.30)⁎⁎⁎
		50–59	1.12 (1.08–1.17)⁎⁎⁎	1.11 (1.05–1.18)⁎⁎⁎	1.22 (1.16–1.28)⁎⁎⁎	1.18 (1.10–1.27)⁎⁎⁎
		60–74	1.10 (1.07–1.14)⁎⁎⁎	1.05 (0.99–1.12)	1.17 (1.11–1.22)⁎⁎⁎	1.08 (0.99–1.17)
		75 plus	1.04 (0.96–1.12)	1.07 (0.97–1.17)	1.06 (0.96–1.18)	1.10 (0.98–1.25)

⁎ 0.01<*p*≤0.05.

⁎⁎ 0.001<*p*≤0.01.

⁎⁎⁎ *p*<0.001

a IQRs=11.9 proximity-weighted outlets per km^2^ for on-sales and 10.6 for off-sales outlets.

b Models adjusted for datazone level income deprivation, and city.
